# Application of natural antioxidants from traditional Chinese medicine in the treatment of spinal cord injury

**DOI:** 10.3389/fphar.2022.976757

**Published:** 2022-10-05

**Authors:** Zhihua Huang, Jingyi Wang, Chun Li, Weihong Zheng, Junyuan He, Ziguang Wu, Jianbang Tang

**Affiliations:** ^1^ Zhongshan Hospital of Traditional Chinese Medicine Affiliated to Guangzhou University of Chinese Medicine, Zhongshan, China; ^2^ Faculty of Chinese Medicine, Macau University of Science and Technology, Macau, China; ^3^ Zhongshan Hospital of Traditional Chinese Medicine, Zhongshan, China

**Keywords:** spinal cord injury, traditional Chinese medicine, oxidative strees, natural medicine, antioxidants

## Abstract

Spinal cord injury (SCI) is a devastating central nervous system disease, caused by physical traumas. With the characteristic of high disability rate, catastrophic dysfunction, and enormous burden on the patient’s family, SCI has become a tough neurological problem without efficient treatments. Contemporarily, the pathophysiology of SCI comprises complicated and underlying mechanisms, in which oxidative stress (OS) may play a critical role in contributing to a cascade of secondary injuries. OS substantively leads to ion imbalance, lipid peroxidation, inflammatory cell infiltration, mitochondrial disorder, and neuronal dysfunction. Hence, seeking the therapeutic intervention of alleviating OS and appropriate antioxidants is an essential clinical strategy. Previous studies have reported that traditional Chinese medicine (TCM) has antioxidant, anti-inflammatory, antiapoptotic and neuroprotective effects on alleviating SCI. Notably, the antioxidant effects of some metabolites and compounds of TCM have obtained numerous verifications, suggesting a potential therapeutic strategy for SCI. This review aims at investigating the mechanisms of OS in SCI and highlighting some TCM with antioxidant capacity used in the treatment of SCI.

## 1 Introduction

Spinal cord injury (SCI) is a neurological injury disease caused by trauma or intraspinal lesions, which often leads to partial or complete loss of sensory and motor functions below the injury segment (Eckert and Martin, 2017). The damage to neuronal structure and function has the irreversible property, leaving SCI patients usually presented with a poor prognosis and a high rate of disability. The severe and long-term physical injury will not only reduce the patients’ quality of life but also result in consequences of serious economic burden on their families (Eli et al., 2021).

The spinal cord contains gray matter and white matter that include nerve cell bodies, along with the ascending tracts and descending tracts. According to the classification of the external physical impact and internal impacts, SCI can be divided into traumatic and non-traumatic spinal cord injury. The former is induced by trauma, such as acute stretch, acute distraction, compression, and axonal shearing (Eckert and Martin, 2017), which is mainly discussed in this review. The latter is often caused by compression of tumors and congenital diseases (Gedde et al., 2019). Degrees of disability can vary from slight sensory abnormality and motor dysfunction to severe paralysis, based on various locations and the traumatic extent of SCI.

The pathophysiological changes of SCI are very complex, so the mechanism of SCI is not well defined. But previous studies have found that oxidative stress (OS) caused by excessive reactive oxygen species (ROS) could aggravate the damage of neurons. Thus, our paper focused on the mechanisms of OS affecting SCI and discussed the etiology of OS from the perspective of traditional Chinese medicine (TCM) theory for SCI patients. Additionally, we highlighted some metabolites and compounds of TCM with antioxidant capacity used in the treatment of SCI, providing the potential treatment therapy of SCI *via* using the natural antioxidants derived from TCM.

## 2 Materials and methods

In this review, an electronic search of published articles published between 2000 and 2021 was conducted from Web of Science, PubMed, Science Direct, Google Scholar, and China National Knowledge Infrastructure (CNKI), with the use of the following keywords: “spinal cord injury, oxidative stress, and traditional Chinese medicine or herbal medicine or Chinese herbal medicine”.

Besides, to ensure the accurate scientific nomenclature for plants in our paper, all names and information on the source species were obtained and validated from Kewscience (http://mpns.kew.org/mpns-portal/) and pharmacopeia of the People’s Republic of China.

## 3 The pathophysiology of spinal cord injury

The pathophysiology of SCI is complicated, which contains acute phase, subacute phase and chronic phase (Supplementary Figure S1). The primary occurrence of SCI results from the initial trauma immediately, including acute stretch, acute distraction, compression, and axonal shearing (Katoh et al., 2019). The primary injury initiated by trauma will progressively cause the secondary injury, and further leads to the lesion of adjacent, uninjured tissue (Anjum et al., 2020). The pathophysiology of secondary injury mainly includes damage to neuronal fiber, blood-spinal cord barrier (BSCB) destruction, excessive free radical production (von Leden et al., 2017), ion imbalances (Garcia et al., 2018), inflammation (Zrzavy et al., 2021) neural cell necrosis and apoptosis (Wang et al., 2019), demyelination (Fan et al., 2018), Among the pathological processes of secondary injury, oxidative stress plays an indispensable and critical role in contributing to a poor microenvironment development, which is considered a hallmark of the secondary injury of SCI (Savikj et al., 2019).

### 3.1 The association between oxidative stress and spinal cord injury

**FIGURE 1:**
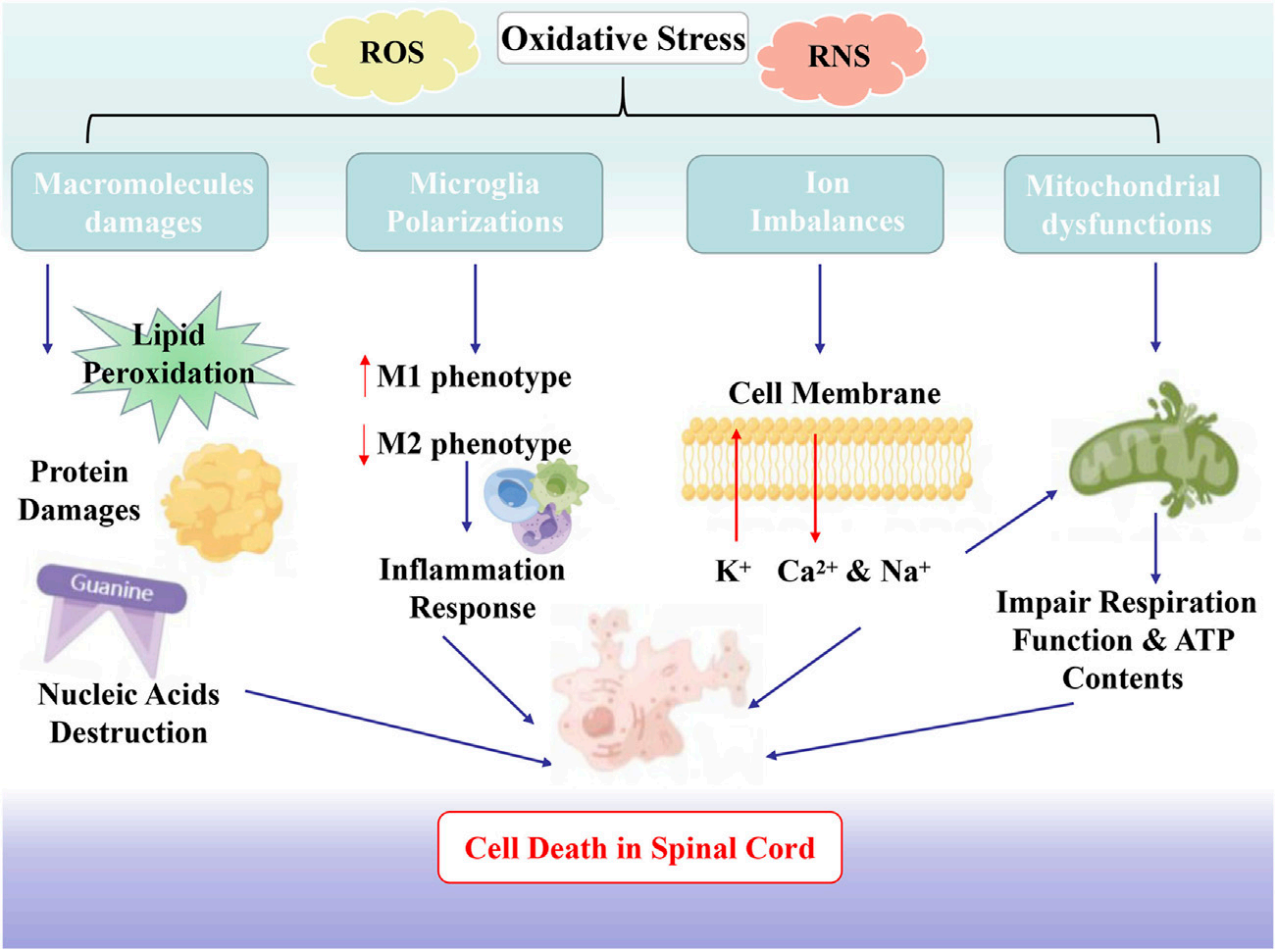
The Association Between Oxidative Stress and SCI. Oxidative stress substantively leads to ion imbalance, lipid peroxidation, inflammatory cell infiltration, mitochondrial disorder, and neuronal dysfunction.

The essence of the OS reaction is the imbalance between oxidative free radicals and antioxidants. Oxidative free radicals include ROS and reactive nitrogen species (RNS) (Fatima et al., 2015). ROS contains superoxide, singlet oxygen, hydroxyl radical, and hydrogen peroxide, mainly generated by the mitochondria and microglia. RNS are also involved in the pathological mechanism of SCI, including nitric oxide (NO) and peroxynitrite (PON). Excessive generation of NO can induce neuronal apoptosis *via* cytotoxic effects (Cadenas, 2018) and PON are the key initiator of the lipids peroxidation (LP), as well protein nitration in the SCI (Hall et al., 2016) (Figure 1).

#### 3.1.1 Oxidative stress causes the damage of biological macromolecules in spinal cord injury

Notably, the spinal cord is very vulnerable to oxidative damage because it contains overabundant polyunsaturated fatty acids that are particularly susceptible to ROS peroxidation (Anjum et al., 2020). Therefore, LP easily occurs after the trauma, which progressively includes three chemical phases, namely initiation, propagation, and termination (Hall et al., 2016). To begin with, highly reactive oxygen radical with the function of electron-snatching, such as -OH, -NO2, or -CO3, has a reaction with the polyunsaturated fatty acid composition of the membrane, such as arachidonic acid, eicosapentaenoic acid, and linoleic acid, causing the damage of membrane integrity. These types of radicals can be considered to have the characteristic called “electrophilic,” which snatches the electron from polyunsaturated fatty acid, leading to quenching of the original radicals whereas the polyunsaturated fatty acid (L), turns to a lipid radical (L•). As for the stage of propagation, L• sets off a chain reaction of constantly producing lipid peroxyl radicals (LOO•) and lipid hydroperoxides (LOOH). When the peroxidizable substrate is depleted or the reaction of lipid radical and another radical or radical scavenger produces other end products, including three carbon-containing malondialdehyde (MDA) and 4-hydroxynonenal (4-HNE) (Hamann and Shi, 2009; Hall et al., 2010), LP greets its termination (Hall et al., 2016). As essential components of membranes, LP can lead to cellular dysfunction, eventually resulting in cell death.

In addition to lipids, proteins are also easy to be damaged by OS, and the damaging effects of OS on proteins are also fundamental factors of the continuous deterioration of SCI. Protein oxidation mainly includes breakage of the polypeptide chain, modification of amino-acid side chains, and protein degradation, as well as the conversed derivatives from protein, which are sensitive to proteolysis (Hawkins and Davies, 2019). The products of LP, MDA, and 4-HNE can compromise the structural and functional integrity of cellular proteins by undergoing a carbonyl ammonia cross-linking reaction to form covalent compounds (Wu et al., 2017). Besides, PON can trigger an exacerbated overload of intracellular Ca^2+^ overload, activating the cysteine protease calpain, which ultimately results in the damage of several cellular targets (Xiong et al., 2007).

Finally, the destructive effects of OS on nucleic acid substances are also not negligible factors. It is well documented that guanine is the most oxidation prone-nucleobase (Singh et al., 2019). When structured in the G-quadruplex entity, guanine can respond particularly towards OS (Singh et al., 2019), principally reflected in the generation of 8-oxoguanine *via* the initiation of 8-oxoguanine glycosylases (Every and Russu, 2013). Overproduced PON can also react with guanine to form 8-nitroguanine (8-NO(2)-G), resulting in the exchange of G and T bases to cause mutagenic damage (Piao et al., 2011). The consequences of base modification are mutations and genomic instability. Currently, the expression of certain microRNAs has been proven to be altered *via* OS. Excessive ROS generation can enhance the expression of miR-200c by negatively regulating the expression of Fas-associated phosphatase-1 (FAP-1), thereby inducing apoptosis (Yu et al., 2015).

#### 3.1.2 Oxidative stress and microglia polarization in spinal cord injury

Microglia, polarized into two phenotypes: pro-inflammatory (M1) phenotype and anti-inflammatory (M2) phenotype, have great significance to the pathophysiological evolution of SCI (Fan et al., 2018). During acute trauma, microglia in the spinal cord tissue are primarily polarized to the M1 phenotype (Kroner et al., 2014). The polarized M1 has close correlations with the release of TNF-α, IL-6, IL-1β, ROS, NO, glutamate, and superoxide, which can trigger inflammation and OS, further initiating cascades of neurotoxic damage of SCI and contributing to apoptosis and necrosis (Anwar et al., 2016; Tang and Le, 2016). On the contrary, M2 phenotype plays critical role in antagonizing the pro-inflammatory responses and promoting neurodegeneration, accompanied by the release of several neurotrophic factors, anti-inflammatory cytokines (IL-4, IL-10, IL-13), and transforming growth factor beta (TGF-β) (Anwar et al., 2016; Tang and Le, 2016). Meanwhile, M2 phenotype can generate Arg1 that competes with iNOS for the arginine substrate and downregulate NO production, thereby reducing the collateral damage of OS to neuronal tissues (Yang et al., 2016). The homeostasis of the local microenvironment depends on the balance between M1 and M2. Therefore, inhibiting M1 or promoting the polarization of M2 may be an effective therapeutic strategy to promote functional recovery after SCI.

#### 3.1.3 Oxidative stress and ion imbalances in spinal cord injury

The imbalance of Na^+^, K^+^, and Ca^2+^ ions caused by OS is a vital mechanism for triggering necrotic cell death and apoptosis. OS significantly aggravates the influx of Ca^2+^ and Na^+^, as well as the decrease of intracellular K^+^ concentrations *via* destroying cell membrane structure, which could disturb ionic homeostasis and leads to necrotic cell death and apoptosis (Vanzulli and Butt, 2015). As an indispensable participator of synaptic transmission, Ca^2+^ plays a critical role in responding to the injuries of the CNS. Ca^2+^-pump ATPase in plasma and endoplasmic reticulum membrane is highly sensitive to OS. LP caused by OS can interfere with the transportation of Ca^2+^ and increase the intracellular Ca^2+^ concentrations *via* inhibiting the activation of Ca^2+^-pump ATPase. At the same time, the inhibition of Na+/K + -pump ATPase caused by OS can increase the accumulation of Na+and further promote the accumulation of Ca^2+^ in cells (Lowry et al., 2020). With the influx of Na^+^ and the alterations of change of osmotic pressure, cytotoxic cellular edema occurs, further promoting intracellular phospholipase activity and intracellular acidosis (Piao et al., 2011).

#### 3.1.4 Oxidative stress and mitochondrial dysfunctions in spinal cord injury

Mitochondrial dysfunction is an important section of the cascade of traumatic cell death after SCI. Following the exacerbation of injury, there is a loss of homeostasis of mitochondria, together with the imbalance of synaptic homeostasis. As energy reservoirs, mitochondria can regulate cytosolic Ca^2+^ ion levels (Turtle et al., 2019). After trauma, the mitochondrial damage is mediated by the elevated Ca^2+^ levels in SCI. Elevated cytosolic Ca^2+^ levels can activate NADH dehydrogenase, increase ATP generation and promote ROS production. The surfeit of Ca^2+^ further disturbs the proton gradient and results in cell swelling or death by opening the mitochondrial permeability transition pores (mPTPs) (Cao et al., 2013). Besides, three PON forms (ONOO−, ONOOCO2, and ONOOH) can deplete stores of mitochondrial antioxidants, leading to protein nitration (Valdez et al., 2000). Excessive PON ulteriorly induced 4-HNE, 3-nitrotyrosine (3-NT), and protein carbonyl content in mitochondrial proteins, further aggravating mitochondrial dysfunction (Sullivan et al., 2007). An extremely low concentration of 4-HNE could significantly impair the respiration function of mitochondria (Xiao et al., 2017). Moreover, NO has been also shown to exist in mitochondria stemming from a NO synthase isoform. Because the neurons are particularly sensitive to energy, preventing the lack of energy prone to cause neurodegeneration is crucial. However, widespread damage to mitochondria can finally and easily cause neuronal death owing to the insufficient energy provided for cell survival (Golpich et al., 2017). Hence, mitochondria are highly regarded as the potential vital target for SCI pharmacological treatment. During the first 6 h after SCI, promoting mitochondrial fusion may even be used as a potential method of improving spinal cord function (Cao et al., 2013).

## 4 Mechanism of oxidative stress in the aetiology of traditional Chinese medicine for spinal cord injury

Therefore, alleviating OS is a critical step in therapeutic intervention in SCI. Seeking the appropriate antioxidant therapy to prevent OS after the injury has become a top priority. With its natural antioxidant capacity, TCM has recently become a novel and promising supplementary treatment. The TCM theory holds that the pathogenesis of SCI is characterized by blood stasis and deficiency of qi. In the theory of TCM, qi is regarded as a very delicate substance with strong vitality. Constituting the human body and maintaining human life activities, the concept of qi is consistent with the adenosine triphosphate (ATP) that is mainly produced in the mitochondrion (Wong and Ko, 2013; Yong et al., 2020). Moreover, Some TCM with the “Qi-invigorating” action has a close link to the safeguarding of mitochondrial function (Kang et al., 2020; Tian et al., 2021). Mitochondrial dysfunction induced by OS resembles the deficiency of qi. The function of qi lies in the power to promote blood flow. When the power of push is insufficient, flowing blood will generate the pathological product, blood stasis. The blockade of blood stasis obstructs the transportation and absorption of nutrients in channels and collaterals, leading to the body not being nurtured, which is consistent with the neurodegeneration caused by the lack of energy owing to the mitochondrial dysfunction induced by OS. The equivalence between qi deficiency and mitochondrial dysfunction provides a modern microbiology perspective for TCM to understand the pathological mechanism. Thus, TCM can treat SCI by supplementing qi, activating blood circulation, and removing blood stasis. Additionally, from the modern perspective, as natural antioxidants, TCM intervenes in SCI by attenuating OS and preventing mitochondrial dysfunction. The overlap of the two theories makes the feasibility of auxiliary treatment of SCI with TCM.

## 5 Therapeutic intervention with traditional Chinese medicine

Attracting a great quantity of attention, TCM has been considered a promising supplementary treatment in recent years. Active herbal extracts, metabolites (Tables 1, 2), traditional botanical drugs (Table 3), and formulas (Table 4) all have shown their effectiveness, playing vital roles in the prevention and treatment of SCI, especially as natural antioxidants.

**TABLE 1:** Therapeutic intervention with active herbal extracts and metabolites.

	Active herbal extracts and metabolites				
Name	Source	Molecular formula	Molecular structure	Mechanisms	Effects on SCI
Quercetin	*Bupleurum chinense* DC [Apiaceae; Bupleuri Radix] and *Morus alba* L. [Moraceae; Mori Folium]	C_15_H_10_O_7_	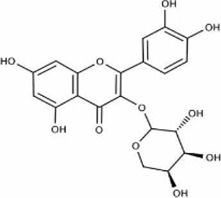	Decreases MDA and iNOS-positive cells, activates ATF2 pathway and suppresses NF-κB and STAT1 pathway	Decreases necroptosis, antioxidant properties
Gastrodin	*Gastrodia elata* Blume [Orchidaceae; Gastrodiae rhizome]	C_13_H_18_O_7_	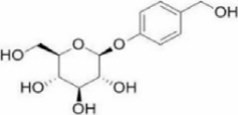	Enhances expressions of Nrf2 and modifies subunit of GCLm and GCLc, decreases ROS and MDA	Recovery of locomotor function and antioxidant properties
Asiatic acid	*Centella asiatica* (L.) Urb. [Apiaceae; Centeliae herba]	C_30_H_48_O_5_	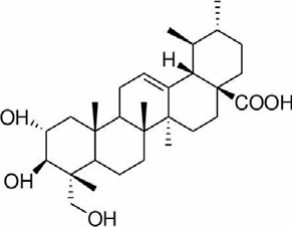	Suppresses myeloperoxidase activity, reduces ROS and MDA and blocks NF-kB/STAT3/ERK pathway	Increases BBB scores and inclined plane test scores, antioxidant effects
Tetramethylpyrazine	*Ligusticum chuanxiong* Hort. [Apiaceae; Chuanxiong Rhizoma]	C_8_H_12_N_2_	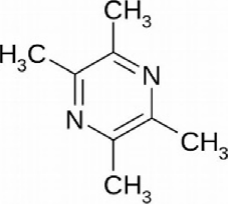	Reduces ROS production and inhibits p53/MAPK pathways	Prevents mitochondria dysfunction, antioxidant effects
Schisandrin B	*Schisandra chinensis* (Turcz) Baill. [Schisandraceae; Schisandrae Chinensis Fructus]	C_23_H_28_O_7_	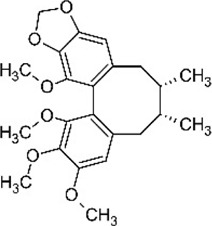	Increases SOD expression and decreases MDA expression	Improves the inclined plate test scores, antioxidant properties
Rosmarinic acid	*Perilla frutescens* (L.) Britton. [Lamiaceae; Perillae Fructus]	C_18_H_16_O_8_	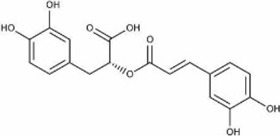	Decreased ROS and activates Nrf2 and HO-1 pathway	Mitigates cytotoxicity and inflammatory injury, antioxidant properties
Allicin	*Allium sativum* L. [Amaryllidaceae; Allii Sativi Bulbus]	C_6_H_10_OS_2_	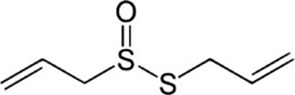	Increases Nrf2 nuclear translocation, inhibits ROS and LP. Inhibit lipid peroxidation by quenching free radicals	Accelerates recovery of motor functions, antioxidant properties
Resveratrol	*Polygonum cuspidatum* Sieb. et Zucc. [Polygonaceae; Polygoni Cuspidati Rhizoma et Radix]	C_14_H_12_O_3_	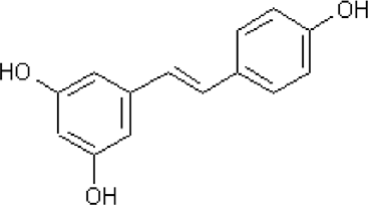	Inhibits the iNOS/p38MAPK pathway and activates Nrf2/HO-1 pathway	Strong antioxidant effects
Crocin	*Crocus sativus L.* [Iridaceae; Croci Stigma]	C_44_H_64_O_24_	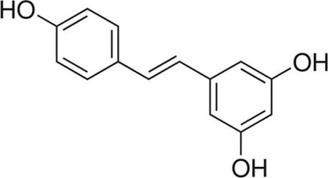	Enhances the expression level of neurotrophic factors in epidermal neural crest stem cells	Promotes spinal cord regeneration
Tetrandrine	*Stephania tetrandra S.Moore* [Menispermaceae; Stephaniae Tetrandrae Radix]	CHNO	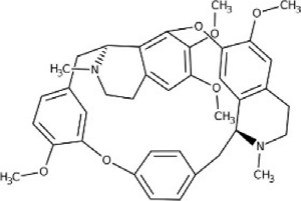	Reduces the production of pro-inflammatory factors and regulates PI3K/AKT/NF-κB pathway	Repairs the integrity of the blood-spinal cord barrier
Lycopene	Tomatoes, watermelons, and pink grapefruits	C_40_H_56_	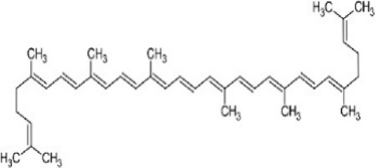	Inhibits MDA and LP	Strong antioxidant effects
Curcumin	*Curcuma longa L*. [Zingiberaceae; Curcumae Longae Rhizoma]	C_21_H_20_O_6_	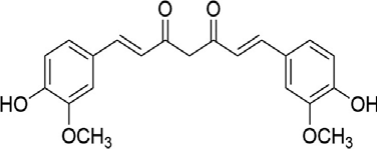	Enhances ability of lymphocytes to resist oxidative stress, increases the SOD content and protects the integrity of mitochondrial membranes	Improves mitochondrial dysfunction and strong antioxidant effects

**TABLE 2:** The specific data of references cited in Section 5.1

Active herbal extracts and monomers	Study type	Experimental subjects	Dose	Duration	Controls
Quercetin	Preclinical trial	40 male rats aged 18 months	*In vivo*: 20 mg/g/d, i.p	10 days	1 ml distilled water both intraperitoneally and subcutaneously
	Preclinical trial	240 adult male Sprague–Dawley rats	*In vivo*: 0.2 mg/kg/d, i.p	14 days	Methylprednisolone 30 mg/kg/d, i.p
Gastrodin	Preclinical trial	60 male Sprang-Dawley rats	*In vivo*: 100 or 200 mg/kg/d, i.p	5 days	Intraperitoneally treated with vehicle
	Preclinical trial	36 New Zealand white rabbits	*In vivo*: 100 mg/kg/d	1 day	Perfuse normal saline 100 mg/kg
Asiatic acid	Preclinical trial	32 adult male Sprague-Dawley rats	*In vivo*: 75 mg/kg	1 day	Vehicle
	Preclinical trial	150 Sprague-Dawley rats	*In vivo*: 30 mg/kg/d or 30 mg/kg/d intragastric injection	3 days	Intragastric injection of vehicle 1 h after SCI
Tetramethylpyrazine	Preclinical trial	Adrenal phaeochromocytoma PC12 cells	*In vitro*: 0.3, 0.6, and 1.2 μM	10, 30, 60, and 120 min	Saline
	Preclinical trial	30 Sprague-Dawley rats	*In vivo*: 30 mg/kg, i.p	30 min before occlusion	Normal saline
Schisandrin B	Preclinical trial	40 adult male Sprague-Dawley rats	*In vivo*: 50 mg/kg, orally	5 days	Physiological saline (0.1 ml/100 g, i.p
Rosmarinic acid	Preclinical trial	60 adult female Sprague-Dawley rats	*In vivo*: 10, 20, and 40 mg/kg, i.p	28 days	Normal saline, i.p
Carnosic acid	Preclinical trial	Adult (8 weeks old) male CF-1 mice	*In vivo*: 0.3, 1.0, and 3.0 mg/kg, i.p	2 days	Saline
	Preclinical trial	Adult (8 weeks old) male CF-1 mice	*In vivo*: 1.0 mg/kg, i.p	2 days	Sulforaphane 5.0 mg/kg
Allicin	Preclinical trial	90 female Sprague-Dawley rats	*In vivo*: 2, 10, and 50 mg/kg/d, i.p	21 days	0.9% NaCl daily
	Preclinical trial	40 adult BALB/c mice	*In vivo*: 1, 5, and 10 mg/kg/d, i.p	7 days	2 ml sterile saline
Resveratrol	Preclinical trial	35 Male Sprague–Dawley rats	*In vivo*: 10 mg/kg, i.p	36 h	1 ml of saline, i.p
	Preclinical trial	42 male Sprague-Dawley rats	*In vivo*:100 mg/kg	48 h	Quercetin 200 mg/kg i.p
Crocin	Preclinical trial	Epidermal neural crest stem cells	*In vitro*: 12.5, 50, 100, 200, 500, 1,000, 1,500, 2,000, and 2,500 µM	72 h	1 mM valproic acid
	Preclinical trial	25 Female Wistar rats	*In vivo*: 150 mg/kg/d, i.p	14 days	Vehicle
Tetrandrine	Preclinical trial	Spinal cord astrocytes	*In vitro*: 0.1, 1, 10, and 20 mM	24 h	PI3K inhibitor LY294002 and NF-κB inhibitor PDTC
	Preclinical trial	48 healthy adult male or female New Zealand white rabbits	*In vivo*: 22.5 mg/kg, i.p	Before Ischemia reperfusion injury	Saline
Lycopene	Preclinical trial	30 adult male SD rats	*In vivo*: 5,10,20 mg/kg/d, i.p	7 days	Saline
Curcumin	Preclinical trial	40 male Wistar rats	*In vivo*: 200 mg/kg, i.p	Immediately after the trauma	1 ml of rice bran oil and 30 mg/kg methylprednisolone sodium succinate
	Preclinical trial	39 male Sprague-Dawley rats	*In vivo*: 40 mg/kg/d, i.p	6 days	Saline

**TABLE 3:** Therapeutic intervention with Chinese herbs.

	Chinese herbs				
Name	Species and source	Components	Picture	Effects in TCM theory	Antioxidant mechanisms
*Salvia miltiorrhiza* Bunge	Lamiaceae; Salviae Miltiorrhizae Radix et Rhizome	Hydrophilic depside derivatives (e.g., danishes, salvianolic acids A–C, E–G, caffeic acid, and ferulic acid) and lipophilic diterpenoids (e.g., tanshinones Ι, ΙΙA, andΙΙB, tanshinoneA, and tanshindiols A and B)	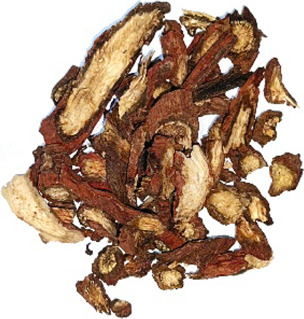	Promotes blood circulation, removes blood stasis, and reduces pain	Improves SOD, decreases the MDA, inhibits MAPK pathways, and preserves CAT activities
*Cistanche deserticola* Ma	Orobanchaceae; Cistanches herba	Total glycosides (TGs, phenylethanoid glycosides, and other glycosides) and oligosaccharides	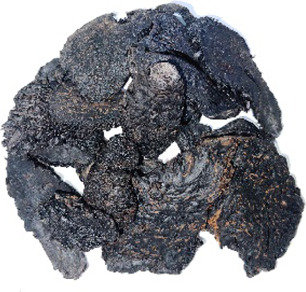	Tonifies yang qi and blood, and moistens the intestines	Reduces p53, IL-6, and TNF-α, decreases MDA, increases SOD, GSH-Px, and CAT, facilitates Nrf-2 nuclear translocation

**TABLE 4:** Therapeutic intervention with traditional formulas.

Name	Effects in TCM theory	Mechanisms
JisuiKang	Removes blood stasis and dredge meridians, reconciles qi and blood	(1) Protects the microstructure of neurons, such as mitochondria, dendritic spine, and endoplasmic reticulum
		(2) Inhibits the expression of the Nogo receptor (NgR) in neurons and reduces the activation of the NgR/RhoA/ROCK signal pathway
		(3) Inhibits the expression of NOS and the content of NO and MDA while improving the activity of SOD
Xuefuzhuyu Decoction	Promotes blood circulation and dissolves stasis	(1) Reduces the content of MDA in the spinal cord and promotes the relief of spinal cord edema
		(2) Improve the content of SOD

### 5.1 Therapeutic intervention with active herbal extracts and metabolites

#### 5.1.1 Quercetin

Quercetin (C_15_H_10_O_7_), is abundant in a variety of plants, fruits, and vegetables, such as onion annd broccoli, as well as *Bupleurum chinense* DC [Apiaceae; Bupleuri Radix] and *Morus alba* L. [Moraceae; Mori Folium], which is one of the major flavonoids that are part of human diets. It plays a role in the prevention of various diseases such as cancer and cardiovascular diseases (Reyes-Farias and Carrasco-Pozo, 2019).

In recent years, it has been revealed that quercetin could protect the injured spinal cord by decreasing oxidative. Aged rats with quercetin (20 mg/kg/d intraperitoneally for 10 days) had a decreased level of MDA and reversed the major degenerative changes, restraining OS (Firgany and Sarhan, 2020). The underlying mechanism may be related to the regulation of p38 mitogen-activated protein kinase (MAPK) and activating transcription factor 2 (ATF2) pathway, thus quercetin antagonized OS. Even compared to the specific p38MAPK inhibitor SB203580, quercetin has stronger effects on enhancing SOD activity and inhibiting MDA in SCI rats. Immunohistochemistry consequences revealed that after quercetin administration (0.2 mg/kg/d intraperitoneally for 14 days), the postoperatively elevated rate of iNOS-positive cells was significantly decreased (Song et al., 2013).

#### 5.1.2 Gastrodin

Gastrodin (C_13_H_18_O_7_) is a phenolic glycoside compound extracted from *Gastrodia elata* Blume [Orchidaceae; Gastrodiae rhizome]. Studies have indicated that gastrodin has various pharmacological effects, such as antihypertensive, lipid-lowering, and anticoagulant, which also beneficial functions in the protection of neurons, by inhibiting OS, regulating immune inflammation, and regulating ion channels (Liu et al., 2018).

Recently, gastrodin has emerged as a potential treatment for SCI. Administration of gastrodin (100 or 200 mg/kg/d intraperitoneally for 5 days) could enhance expressions of nuclear factor (erythroid-derived 2)-like 2 (Nrf2), modified subunit of *γ*-glutamylcysteine ligase (GCLm), and catalytic subunit of *γ*-glutamylcysteine ligase (GCLc), and subsequently improve the OS and recovery of locomotor function, as illustrated by the accumulation of Basso-Beattie-Bresnahan (BBB) scores (Du et al., 2016). Similarly, the spinal cord ischemia-reperfusion injury model by blocking the abdominal aorta under the renal artery and intervening with gastrodin showed an increase in SOD, glutathione peroxidase (GSH-Px), and total antioxidant capacity (T-AOC), as well as the decrease in ROS and MDA. Simultaneously, the reduction of mitochondrial swelling (MSD) also confirmed the effects of gastrodin (100 mg/kg/d intraperitoneally for 1 day) on protecting spinal cord ischemia-reperfusion injury by promoting the antioxidant capacity of spinal cord mitochondria and inhibiting the inflammatory response to the injury (Fang et al., 2016).

#### 5.1.3 Asiatic acid

Asiatic acid (AA, C_30_H_48_O_5_) is a naturally occurring pentacyclic triterpenoid, which is found mainly in the *Centella asiatica* (L.) Urb. [Apiaceae; Centeliae herba]. It has been reported that AA has broad-spectrum anticancer abilities.

Current research has shown that AA can be used to protect damaged spinal cord by reducing OS. AA treatment (75 mg/kg/d intraperitoneally for 1 day) significantly increased BBB scores and inclined plane test scores that were reduced by acute SCI. In addition, AA suppressed myeloperoxidase activity and reduced the levels of pro-inflammatory cytokines, ROS, and MDA, while increasing superoxide dismutase activity and glutathione production (Gurcan et al., 2017). Additionally, intragastric injection with AA (30 mg/kg or 75 mg/kg) was demonstrated that could lead to the downregulation of ROS, MDA, and tumor necrosis factor-alpha, while upregulating superoxide dismutase activity and glutathione production, which may be related to the inhibition of NLRP3 inflammasome pathway and the activation of Nrf2 and HO-1 (Jiang et al., 2016).

#### 5.1.4 Tetramethylpyrazine

Tetramethylpyrazine (TMP, C_8_H_12_N_2_), a natural alkaloid which is isolated from the Chinese botanical drug *Ligusticum chuanxiong* Hort*.* [Apiaceae; Chuanxiong Rhizoma], has been found to have antioxidant and anti-inflammatory effects (AlKreathy et al., 2020).

Previous study reported the TMP treatment (30 mg/kg, intraperitoneally 30 min before occlusion) decreased the expression level of proinflammatory cytokines TNF-α and IL-1β, and inhibited the activation of NF-κB in spinal cord ischemia rats (Fan et al., 2011). Recently, TMP and monosialotetrahexosylganglioside (GM1) have been effectively used in the treatment of SCI. It is well documented that Selenium nanoparticles (SeNPs) have excellent antioxidant activity. As a novel multi-functionalized SeNPs, SeNPs@GM1/TMP, being loaded with TMP/GM1 and decorated with polysaccharide-protein complex (PTW)/PG-6 peptide, showed a strong protective effect against apoptosis after SCI. SeNPs@GM1/TMP (0.3, 0.6 and 1.2 μM) attenuated ROS overproduction *via* inhibiting p53 and MAPK in concentration dependent, thus preventing mitochondria dysfunction (Rao et al., 2019).

#### 5.1.5 Schisandrin B

Schisandrin B (Sch B, C_23_H_28_O_7_), a dibenzocyclooctadiene lignan, is one of the most abundant active dibenzocyclooctadiene derivatives found in the *Schisandra chinensis* (Turcz.) Baill. [Schisandraceae; Schisandrae Chinensis Fructus]. Several studies had shown that Sch B has anti-fibrosis effects (Cuiqiong et al., 2020) and has a protective effect on myocardial injury (Zhang et al., 2017).

Currently, Xin et al. (2017) found that Sch B (50 mg/kg/day orally for 5 days) reduced the inflammatory response and inhibited OS in SCI model rats. ELISA kits showed that Sch B significantly increased SOD expression and decreased MDA expression levels compared with the untreated SCI group, which may be related to the inhibition of the P53 signaling pathway. Besides, Sch B improved the maximum angle of inclined plate test, behavioral examination scores, and inhibited spinal cord water content in rats with SCI.

#### 5.1.6 Rosmarinic acid

Rosmarinic acid (RA, C_18_H_16_O_8_) exists in *Perilla frutescens (L.)* Britton. [Lamiaceae; Perillae Fructus]. RA was proved with multiple biological activities, including, anti-inflammatory, neuroprotective, and antiangiogenic abilities (Colica et al., 2018).

In the animal experiment conducted by Ma et al. (2020), they found that RA notably upregulated the activities of CAT, SOD and GSH-Px and downregulated the MDA levels, indicating the attenuation of SCI-induced oxidative damage. Among the three groups designed in their study, the highest content administration of RA (40 mg/kg) showed significantly highest levels of SOD, CAT and GSH-Px than groups with 20 mg/kg and 10 mg/kg RA (*p* < 0.05). Besides, Treatment with RA could remarkably increase Nrf2 and HO-1 levels to ameliorate the increase in oxidative injury and apoptosis induced by H_2_O_2._ Meanwhile, the activation of the Nrf2/HO-1 pathway further amplified the inhibition of the NF-κB pathway, mitigating LPS-induced cytotoxicity and inflammatory injury.

#### 5.1.7 Allicin

Allicin (C_6_H_10_OS_2_), a thioester of sulfenic acid, is a natural antioxidant found in *Allium sativum* L. [Amaryllidaceae; Allii Sativi Bulbus]. Studies have shown that allicin has a good effect on a variety of diseases, such as bone diseases (Ding et al., 2016; Yang et al., 2020; Li et al., 2021) and neurodegenerative diseases (Zhang et al., 2018).

It is noteworthy that allicin played an important therapeutic role in SCI by inhibiting OS. SCI rats administrated with allicin showed better histological outcomes and accelerating recovery of motor functions, which may be related that allicin increased Nrf2 nuclear translocation in SCI rats (Lv et al., 2015). Additionally, the protective effects of allicin on antioxidation in SCI rats were dependent on the elevated production of NADH and inhibited levels of ROS (Wang and Ren, 2016). In the SCI model induced by glutamate, allicin was also shown to attenuate the release of lactate dehydrogenase (LDH), and inhibit OS by the mediation of heat shock protein 70 (HSP70)/inducible nitric oxide synthase (iNOS) pathway (Wang and Ren, 2016). Collectively, allicin may serve as a new therapeutic strategy for spinal cord injury (Liu et al., 2015).

#### 5.1.8 Resveratrol

Resveratrol (C_14_H_12_O_3_) is a kind of natural non-flavonoid polyphenol found in many vegetables and fruits, mainly in grapes and *Polygonum cuspidatum* Sieb. et Zucc. [Polygonaceae; Polygoni Cuspidati Rhizoma et Radix]. Numerous studies have shown that resveratrol can serve as a good antioxidant, improving overall health by inhibiting OS (Wang et al., 2013; Chen et al., 2020; Recalde et al., 2020).

In the animal experiment conducted by Fu et al., resveratrol (10 mg/kg intraperitoneally) functioned as a strong antioxidant to protect the ischemia-damaged spinal cord by inhibiting the iNOS/p38MAPK signaling (Fu et al., 2018). Moreover, resveratrol (100 mg/kg intraperitoneally) was found that it had a good preventive effect on secondary injury caused by SCI (Çiftçi et al., 2016).

#### 5.1.9 Crocin

Crocin (C_44_H_64_O_24_), belonging to apocarotenoid glycosides, is the main colorant and bioactive ingredient in *Crocus sativus* L. [Iridaceae; Croci Stigma]. Studies have shown that crocin has attracted much attention in the field of food and cosmetics. It can be used to produce functional products and as an anti-aging cosmetic agent for the skin (Fagot et al., 2018). Despite being used in dermatology, the results of Abdulkareem Aljumaily et al. (2021) verified that it can be used as a heart-protective agent for patients with cancers.

In recent years, crocin also has been found to have a significant effect on SCI. A recent investigation demonstrated that crocin may have therapeutic effects on SCI by enhancing the expression level of neurotrophic factors in epidermal neural crest stem cells (Baharvand et al., 2020). SCI rats treated with crocin (150 mg/kg/d intraperitoneally for 2 weeks) presented a notably reduced level of calcitonin gene-related peptide than the control group (Karami et al., 2013). In a word, crocin can greatly promote spinal cord regeneration and is an effective drug for treating SCI (Terraf et al., 2017).

#### 5.1.10 Tetrandrine

Tetrandrine (CHNO) is an alkaloid, which mainly exists in the dry roots of *Stephania tetrandra* S. Moore [Menispermaceae; Stephaniae Tetrandrae Radix]. In recent years, studies have investigated that it can lessen lung damage, having a potential role in the treatment of asthma (Lin et al., 2019; Lin et al., 2020).

As for the aspect of tetrandrine treating SCI, scholars found tetrandrine was capable to protect damaged spinal cord astrocytes in rats and diminished the accumulation of IL-1β, IL-6, and TNF-α, which may be related to PI3K/AKT/NF-κB signaling pathway (Bao et al., 2016). After being intravenously injected with tetrandrine (22.5 mg/kg), New Zealand white rabbits with ischemia-reperfusion injury of the spinal cord showed a notable repair of the integrity of the blood-spinal cord barrier, related to that tetrandrine could change the BCL-2/Bax ratio and reduce the production of pro-inflammatory factors (Pu et al., 2020).

#### 5.1.11 Lycopene

Lycopene (C_40_H_56_), an open-chain unsaturated structure, is the most important carotenoid in human plasma. Tomatoes, watermelons and pink grapefruits, are all rich sources of lycopene (Grabowska et al., 2019). As a functional pigment, lycopene has a variety of biological effects, among which the antioxidant effect has received extensive attention.

In recent years, numerous studies have found that lycopene could inhibit the production of MDA, and effectively resist ROS-mediated lipid peroxidation, thereby reducing the secondary damage of free radicals to spinal blood vessels and nerve cells. Animal experiments showed that lycopene also had an antioxidant effect on mitochondrial dysfunction in SCI rats. The experimental data showed that the mtDNA content and function-related genes-cytochrome b and mitochondrial organism’s function-Tfam of the SCI group significantly decreased. However, these changes were significantly hyperpolarized after intraperitoneally injecting lycopene (Hu et al., 2017). In addition, the outstanding effects of lycopene were directly proportional to the use time.

#### 5.1.12 Curcumin

Curcumin (C_21_H_20_O_6_) is a natural polyphenol compound, which is extracted from traditional Chinese medicine *Curcuma longa* L. [Zingiberaceae; Curcumae Longae Rhizoma]. Studies have found that curcumin’s properties of anti-inflammatory, anti-glial cells, and antitumor activity (Vollono et al., 2019) (Barua and Buragohain, 2021).

In recent years, research results inferred that curcumin may play a therapeutic effect in SCI. In an animal experiment (Cemil et al., 2010), researchers applied curcumin (200 mg/kg/d intravenously for 1 day) to treat aneurysm clamp SCI and detected enzyme changes in the tissue. Their BBB score confirmed the recovery of nerve function after administrating with curcumin. Lin and his colleagues (Lin et al., 2011) established the spinal cord hemisection injury model and observed that curcumin (40 mg/kg/d intravenously for 6 days) could improve the motor function of SCI rats. The mechanism may be related to that curcumin increased the SOD content in spinal cord tissue, thereby promoting the production of GSH in astrocytes, improving mitochondrial dysfunction and lipid peroxidation, along with protecting the integrity of mitochondrial membranes.

### 5.2 Therapeutic intervention with Chinese botanical drugs

#### 5.2.1 Salvia miltiorrhiza Bunge

As one of the most famous Chinese botanical drugs, Danshen, namely *Salvia miltiorrhiza* Bunge [Lamiaceae; Salviae Miltiorrhizae Radix et Rhizome] has the effects of promoting blood circulation, removing blood stasis, and reducing pain in TCM theory. Danshen has plenty of components, majorly including hydrophilic depside derivatives (e.g., danishes, salvianolic acids A–C, E–G, caffeic acid, and ferulic acid) and lipophilic diterpenoids (e.g., tanshinones Ι, ΙΙA, andΙΙB, tanshinoneA, and tanshindiols A and B).

Recently, studies found that many components of Danshen could play a critical antioxidant role in improving SCI. Dihydrotanshinone I (DI) could alleviate the pathological damage to the spinal cord and promote neuronal functional recovery by suppressing iNOS, and total oxidant status levels, while improving the (total antioxidant status) TAS level. Moreover, the experiment revealed that the HMGB1/TLR4/NOX4 pathway may participate in the effects of DI on SCI (Yu and Qian, 2020). Intraperitoneal administration with tanshinone IIA (TIIA) inhibited OS by significantly rescuing the activity of SOD and decreasing the MDA. Notably, TIIA had strong analgesic actions *via* inhibiting MAPKs pathways to depress the activation of microglial and immune responses (Cao et al., 2015). Salvianolic acid B(SalB) In the SCI rat models, groups administered with SalB markedly preserved the activities of SOD and CAT, playing an antioxidant role. (Fu et al., 2014).

#### 5.2.2 Cistanche deserticola ma

*Cistanche deserticola* Ma [Orobanchaceae; Cistanches herba] is a commonly used drug for tonifying yang qi and blood, and moistening the intestines in TCM theory. Modern medicine has proved that *Cistanche deserticola* is effective for cardiovascular and cerebrovascular diseases (Wang et al., 2020). A considerable number of components of *Cistanche deserticola* were shown to be antioxidants, including total glycosides (TGs, phenylethanoid glycosides, and other glycosides) and oligosaccharides.

It was reported that oligosaccharides from *Cistanche deserticola* significantly altered LP, GSH, superoxide dismutase, acetylcholine esterase, catalase, nitric acid, acetylcholine esterase, and ROS in the SCI rats. Extract supplementation of oligosaccharides reduced mRNA expression levels of iNOS, p53, IL-6, TNF-α and cyclooxygenase-2 more than 20%, showing effective effects against inflammation, apoptosis, and OS (Zhang et al., 2019). Moreover, according to the experimental results of Wang et al. (2020) TGs could significantly decrease MDA levels, while increasing antioxidant activities, such as SOD, GSH-Px, and CAT, *via* remarkably facilitating Nrf-2 nuclear translocation. Additionally, phenylethanoid glycosides, an extract of *Cistanche deserticola* also had the ability to enhance SOD activity and decreasing MDA content (Xuan and Liu, 2008).

### 5.3 Therapeutic intervention with traditional formulas.

#### 5.3.1 JisuiKang

JSK, a TCM formula derived from classic prescriptions Buyang Huanwu Decoction, is composed of *Plantago asiatica* L. [Plantaginaceae; Plantaginis Semen] 15 g, *Paeonia lactiflora* Pall. [Paeoniaceae; Paeoniae Radix Rubra] 12 g, *Ligusticum chuanxiong* Hort*.* [Apiaceae; Chuanxiong Rhizoma] 10 g, *Rheum palmatum* L. [Polygonaceae; Rhei Radix et Rhizoma] 10 g, *Angelica sinensis* (Oliv.) Diels [Apiaceae; Angelicae Sinensis Radix] 12 g, Salvia miltiorrhiza Bunge [Lamiaceae; Salviae Miltiorrhizae Radix et Rhizome] 20 g, *Poria cocos* (Schw.) Wolf [Polyporaceae; Poria] 10 g, *Magnolia officinalis* Rehd. et Wils. [Magnoliaceae; Magnoliae Officinalis Cortex] 10 g; *Astragalus mongholicus* Bunge [Fabaceae; Astragali Radix] 30 g, *Cistanche deserticola* Ma [Orobanchaceae; Cistanches herba] 10 g, *Eupolyphaga sinensis* Walker [Corydidae; Eupolyphaga Steleophaga] 10 g, *Scolopendra subspinipes mutilans* L. Koch [Scolopendridae; Scolopendra] 10 g, *Epimedium brevicornu* Maxim. [Berberidaceae; Epimedii Folium] 10 g**,** *Alpinia oxyphylla* Miq. [Zingiberaceae; Alipiniae Oxyphyllae Fructus] 10 g, *Alisma orientale* (Sam.) Juzep. [Alismataceae; Alismatis Rhizoma] 10 g, *Citrus aurantium* L. [Rutaceae; Aurantii Fructus Immaturus] 10 g. Many clinical tests on SCI patients have demonstrated that JSK has satisfactory clinical efficacy (Guo et al., 2017). JSK could improve the motor function of SCI rats by protecting the microstructure of neurons, such as mitochondria, dendritic spine, and endoplasmic reticulum. Moreover, JSK inhibited the expression of the Nogo receptor (NgR) in neurons and reduced the activation of the NgR/RhoA/ROCK signal pathway to improve the motor function of SCI rats. These effects indicated the clinical values of JSK as a potential nerve regeneration agent (Wu et al., 2020).

As for the aspects of antioxidation, JSK could inhibit the expression of NOS and decrease the content of NO and MDA, while improving the activity of SOD. Thus, JSK directly inhibited the process of LP after SCI, as well as weakened the secondary degeneration and necrosis of the spinal cord caused by free radicals. On the other hand, JSK could gradually enhance the activity of endogenous antioxidants and enhance the scavenging effects of free radicals (Zhou et al., 2009). Moreover, in the clinical trial including 84 SCI patients, Wang et al. (2008) demonstrated that JSK treatment had significantly beneficial effects in improving kinetic score, grades of spinal injury and effectiveness of the treatment (*p* < 0.05). Clinical trial including 68 SCI patients demonstrated that the effective rate of JSK treatment was 94.3%, which may be related to the inhibited expressions of GFAP and CSPG proteins (Shao, 2020).

#### 5.3.2 Xuefuzhuyu decoction

Originated in Qing Dynasty, XFZYD is considered one of the most significant decoctions for promoting blood circulation and dissolving stasis to treat cardiovascular and cerebrovascular diseases. The abundant meta-analysis, clinical trials, and animal experiments showed that XFZYD had effects on treating hyperlipidemia (Liao et al., 2014), coronary heart disease (Liao et al., 2014), liver fibrosis (Zhou et al., 2014), and so on. Composed of 11 kinds of traditional botanical drugs, including *Angelica sinensis* (Oliv.) Diels [Apiaceae; Angelicae Sinensis Radix] 9 g, *Rehmannia glutinosa* Libosch. [Orobanchaceae; Rehmanniae Radix] 9 g, *Prunus persica* (L.) Batsch [Rosaceae; Persicae Semen] 12 g, *Citrus aurantium* L. [Rutaceae; Aurantii Fructus] 6 g, Paeonia lactiflora Pall. [Paeoniaceae; Paeoniae Radix Rubra] 6 g, *Carthamus tinctorius* L. [Asteraceae; Carthami Flos] 9 g, *Ligusticum chuanxiong* Hort. [Apiaceae; Chuanxiong Rhizoma] 5 g, *Bupleurum chinense* DC. [Apiaceae; Bupleuri Radix] 3 g, *Glycyrrhiza uralensis* Fisch. [Fabaceae; Glycyrrhizae Radix et Rhizoma] 6 g, *Platycodon grandiflorum* (Jacq.) A. DC. [Campanulaceae; Platycodonis Radix] 5 g, *Achyranthes bidentata* Blume [Amaranthaceae; Achyranthis Bidentatae Radix] 9 g, XFZYD was found to be beneficial for the treatment of SCI by attenuating OS.

XFZUD could promote the microcirculation reperfusion of spinal cord stasis, reduce the content of MDA in the spinal cord and promote the relief of spinal cord edema, so as to protect the injured spinal cord (Liu et al., 2006). Meanwhile, a current study showed that the content of SOD in XFZYD containing serum group was significantly higher than that in the injury control group and blank serum group (Liu et al., 2017). Besides, the level of SOD in the XFZYD group was similar to that in the neurotrophic factor protection (NFP) group, and this similitude also existed in the cell survival rate of these two groups (XFZYD group = 0.529 ± 0.010, NFP group = 0.548 ± 0.056) (Liu et al., 2017).

## 6 Conclusion

The complicated pathophysiologic mechanisms contained in SCI lead to the existing circumstances that fully restorative treatments of SCI do not exist. Among the various pathophysiologic mechanisms, OS plays a vital role in secondary injury, triggering a series of free-radical-mediated damages, including damages to biological macromolecules, ion imbalances, and mitochondrial dysfunctions. Thus, lessening OS may become an effective therapeutic strategy for SCI.

From classical TCM theory, the mentioned active herbal extracts, metabolites, traditional botanical drugs, and formulas can treat SCI *via* invigorating Qi, activating blood circulation, and removing blood stasis, which is in line with our above-mentioned TCM treatment principles for SCI. On the other hand, from the modern perspective, the mentioned TCM intervene in SCI by suppressing M1, enhancing SOD activity, decreasing MDA levels, promoting mitochondrial functions, and other pathways to attenuate the impacts of OS on SCI. In the treatment of SCI, traditional botanical drugs and formulas used for thousands of years, have the advantages of synergistic effect and multitarget action, which are more in line with the medication law of TCM theory. However, “Drug-Drug Interaction” (DDI) occurs in the traditional botanical drugs and formulas owing to their numerous compounds, which may cause the complication of predicting and preventing adverse reactions. On the contrary, active herbal extracts and metabolites, new concepts proposed in modern medicine compared to traditional botanical drugs and formulas, have the superiority of simple composition, direct targeting, high safety, and clear effects, avoiding the problems of adverse reactions. Therefore, active herbal extracts and metabolites with scavenging capacity may be more suitable to be the supplementary treatment to improve SCI, particularly resveratrol and quercetin.

However, the investigations on the application of TCM in SCI are generally published in low-impact journals, suggesting that this field of study is still in the initial stages. Additionally, promising results have been widely verified in animal models, instead of the implementation in human clinical applications, which makes us more aware of the obstacles and limitations of TCM in SCI. Therefore, in-depth studies in this study are extremely necessary. Accordingly, we make the following three recommendations and perspectives for subsequent research in this area: 1) to comprehensively understand traditional botanical drugs and formulas, more clinical investigations need to be done, including underlying possible therapeutic mechanisms, the best route of administration, dosage, and timing. 2) combine the active herbal extracts and metabolites with emerging nanotechnology or modern tissue scaffold therapies, improve the effect of nerve function repair and reconstruction after SCI. 3) in-depth research on the traditional theory of TCM and the mechanism of antioxidants in TCM is needed to reveal the modern mechanism of the traditional efficacy of benefiting qi in TCM theory, thereby identifying appropriate treatments for SCI within the TCM treatment protocol.

## Glossary

3- NT: 3-nitrotyrosine
4- HNE: 4-hydroxynonenal
AA: asiatic acid
ATF2: activating transcription factor 2
ATP: adenosine triphosphate
ATRA: all-trans retinoic acid
BBB: Basso-Beattie-Bresnahan
BSCB: blood-spinal cord barrier
CA: Carnosic acid
DDI: Drug-Drug Interaction
EGb: Ginkgo biloba extract
EGCG: Epigallocatechin gallate
FAP-1: Fas-associated phosphatase-1
GSH-Px: glutathione peroxidase
IRI: ischemia-reperfusion injury
iROS: intracellular reactive oxygen species
JSK: JisuiKang
LP: lipid peroxidation
MAPK: mitogen-activated protein kinase
MDA: malondialdehyde
NO: nitric oxide
OS: oxidative stress
PON: peroxynitrite
Q- PCR: Quantitative real-time polymerase chain reaction
RA: Rosmarinic acid
RNS: reactive nitrogen species
ROS: reactive oxygen species
Sch B: Schisandrin B
SCI: Spinal cord injury
SeNPs: Selenium nanoparticles
T- AOC: total antioxidant capacity
TCM: traditional Chinese medicine
TMP: Tetramethylpyrazine
XFZYD: Xuefuzhuyu Decoction
