# Supplementary figures and images for: Application of natural antioxidants from traditional Chinese medicine in the treatment of spinal cord injury
Source: Front Pharmacol. 2022 Oct 5;13:976757. doi: 10.3389/fphar.2022.976757 (PMC9579378; doi:10.3389/fphar.2022.976757)

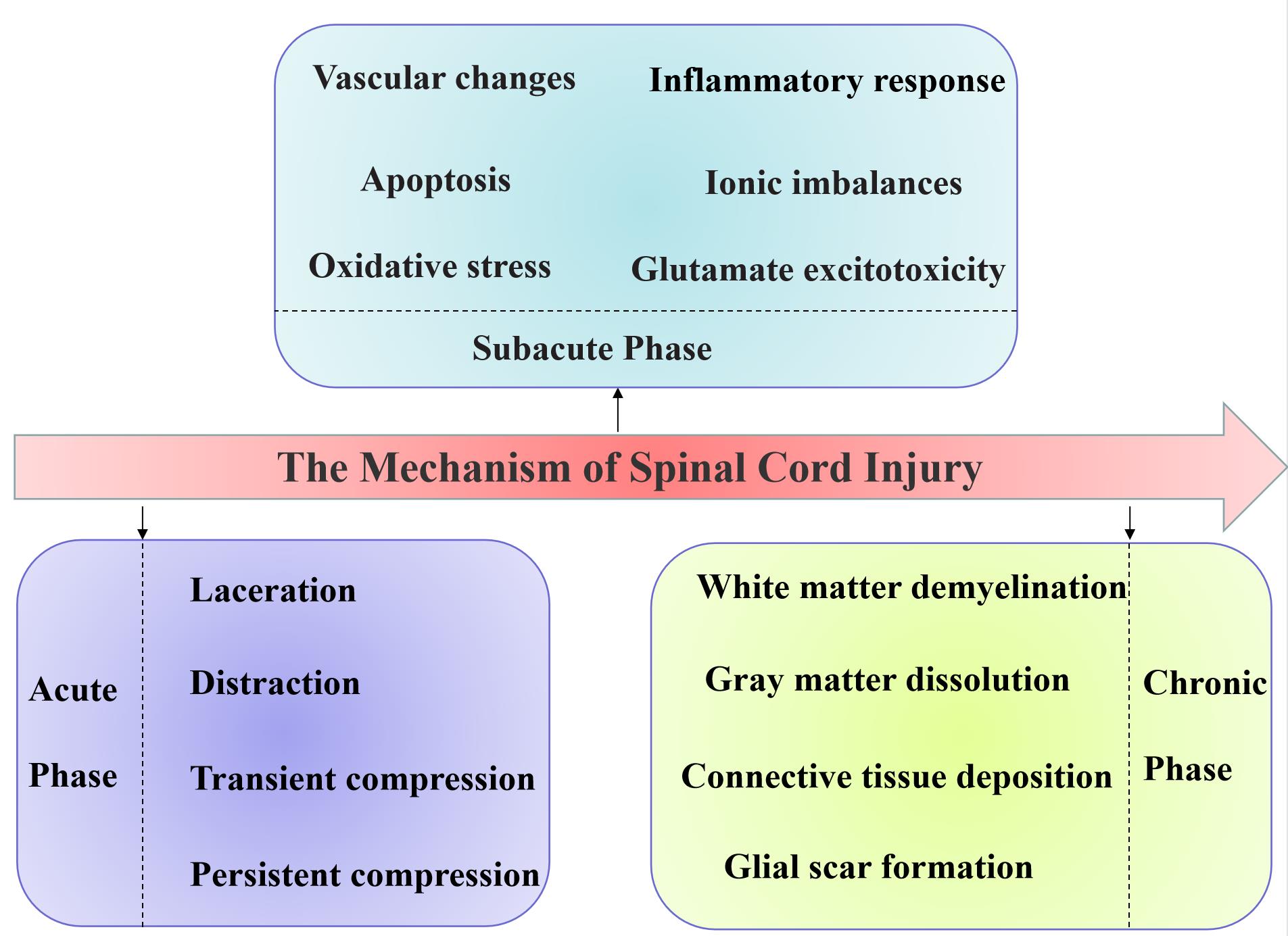

Supplement: Supplementary file 1 [file Image1.JPEG]
